# Breast implant surface topography triggers a chronic-like inflammatory response

**DOI:** 10.26508/lsa.202302132

**Published:** 2024-02-21

**Authors:** Valeriano Vinci, Cristina Belgiovine, Gerardus Janszen, Benedetta Agnelli, Luca Pellegrino, Francesca Calcaterra, Assunta Cancellara, Roberta Ciceri, Alessandra Benedetti, Cindy Cardenas, Federico Colombo, Domenico Supino, Alessia Lozito, Edoardo Caimi, Marta Monari, Francesco Maria Klinger, Giovanna Riccipetitoni, Alessandro Raffaele, Patrizia Comoli, Paola Allavena, Domenico Mavilio, Luca Di Landro, Marco Klinger, Roberto Rusconi

**Affiliations:** 1 IRCCS Humanitas Research Hospital, Rozzano, Italy; 2 Department of Biomedical Sciences, Humanitas University , Pieve Emanuele, Italy; 3 Department of Clinical, Surgical, Diagnostics and Pediatric Sciences, University of Pavia , Pavia, Italy; 4 Department of Aerospace Science and Technology, Politecnico di Milano, Milan, Italy; 5 Department of Medical Biotechnologies and Translational Medicine, University of Milan , Milan, Italy; 6 Department of Health Sciences, University of Milan , Milan, Italy; 7 Fondazione IRCCS Policlinico San Matteo, Pavia, Italy

## Abstract

Breast implants are extensively employed for both reconstructive and esthetic purposes. However, the safety of breast implants with textured surfaces has been questioned, owing to a potential correlation with anaplastic large-cell lymphoma and the recurrence of breast cancer. This study investigates the immune response elicited by different prosthetic surfaces, focusing on the comparison between macrotextured and microtextured breast implants. Through the analysis of intraoperatively harvested periprosthetic fluids and cell culture experiments on surface replicas, we demonstrate that macrotextured surfaces elicit a more pronounced chronic-like activation of leucocytes and an increased release of inflammatory cytokines, in contrast to microtextured surfaces. In addition, in vitro fluorescent imaging of leucocytes revealed an accumulation of lymphocytes within the cavities of the macrotextured surfaces, indicating that the physical entrapment of these cells may contribute to their activation. These findings suggest that the topography of implant surfaces plays a significant role in promoting a chronic-like inflammatory environment, which could be a contributing factor in the development of lymphomas associated with a wide range of implantable devices.

## Abstract (precis)

This study explores the impact of breast implant surface topographies on immune responses, using analyses of periprosthetic fluids and in vitro cultures on model surface replicas, revealing that macrotextured implants significantly induce chronic-like inflammatory reactions.

## Introduction

Breast cancer remains the most prevalent cancer among women globally, with over 2 million new cases reported in 2020, marking an 11.7% increase from the previous year, as per the Global Cancer Observatory (https://gco.iarc.fr/today/home). In the realm of breast cancer treatment, breast reconstruction has become a vital component, offering not only physical restoration but also significant psychological and esthetic benefits for women undergoing mastectomy. In recent years, postmastectomy breast reconstruction has decisively shifted from autologous procedures to implant-based reconstruction, mostly linked to the advantages in terms of minor complications, faster recovery, and reduced healthcare costs (Davis et al, 2020). Furthermore, breast implants are also extensively used in cosmetic surgical procedures. The initial generation of breast implants featured smooth surfaces. However, to prevent potential risks of implant displacement and rotation, and to lower the incidence of capsular contracture, the development and use of implants bearing different types of textured surfaces have gained widespread acceptance (Barnsley et al, 2006; Maxwell et al, 2014). Manufacturers typically classify breast implants based on the characteristic scale of their textured surface features into three categories: smooth (<10 μm), microtextured (10–50 μm), and macrotextured (>50 μm) (https://www.iso.org/standard/63973.html).

The safety of breast implants, particularly in relation to their macrotextured surface, has been questioned since 1997, after the first reported case of breast implant–associated anaplastic large-cell lymphoma (BIA-ALCL) (Keech & Creech, 1997). BIA-ALCL, a non-Hodgkin lymphoma of T-cell origin, was classified as a hemato-lymphoid neoplasm by the World Health Organization (WHO) in 2017 (Arber et al, 2016). Despite the challenge of quantifying an accurate risk assessment because of limited global reporting and incomplete sales data, breast implants have been inserted into the list of agents with high priority for evaluation by the International Agency for Research on Cancer (IARC) for inclusion in their monographs on carcinogenic risks to humans (Srinivasa et al, 2017; Clemens et al, 2018; Marra et al, 2020). In response to safety concerns, France has specifically banned macrotextured devices and nearly 40 different countries have restricted the use of Allergan Biocell breast implants (Maxwell et al, 2014). Allergan’s salt loss manufacturing technique, which creates a notably coarse macrotextured surface, is designed to enhance tissue integration and esthetic outcomes. Yet, despite the extremely common use of textured breast implants, there is very limited knowledge on the correlation between implant surface topography and adverse effects in patients.

Albeit genetic factors may contribute to the onset of this lymphoma (Tevis et al, 2019; Laurent et al, 2020), there is growing evidence that the chronic inflammatory state associated with textured prostheses plays a role in fostering a pro-tumoral environment (Mempin et al, 2021; Mankowski et al, 2022). Recent animal studies involving mice and rabbits have highlighted a direct correlation between the inflammation predominantly mediated by T cells and the roughness of the implant surface (Doloff et al, 2021). The microenvironment of BIA-ALCL has been further associated to an abundance of T helper 17 (Th17) CD4^+^ cells, which are stimulated by cytokines to enhance the inflammatory response, and T-regulatory (Treg) CD4^+^ cells, which serve to suppress the immune response. This setting can be described as a pro-inflammatory milieu with chronic T-cell stimulation, as evidenced by CD30 expression (Wolfram et al, 2012). Other proposed etiopathological hypotheses for BIA-ALCL in the context of textured implants (particularly macrotextured) include mechanical degradation because of friction and chronic inflammation induced by bacterial biofilm (Bewtra et al, 2022; Alessandri-Bonetti et al, 2023). Despite these associations, a direct causal link between implant texturization and tumor development in BIA-ALCL has yet to be established.

In this study, our objective was to investigate the differential microbial contamination and immune responses elicited by macrotextured and microtextured breast implant surfaces. We hypothesized that macrotextured surfaces would provoke a more pronounced inflammatory response than microtextured surfaces, potentially playing a role in the pathogenesis of conditions such as BIA-ALCL. To explore this hypothesis, we conducted an extensive analysis of periprosthetic fluids collected from patients implanted with breast implants having different surface textures. Our approach encompassed examining the bacterial load, profiling immune cell populations, and analyzing the inflammatory cytokine landscape. Our findings indicated a chronic-like inflammatory environment associated with macrotextured implants. Furthermore, in vitro experiments performed culturing healthy donor-derived peripheral blood mononuclear cells (PBMCs) on model surfaces mimicking the characteristics of both microtextured and macrotextured implants corroborated our initial observations, reinforcing the conclusion that the texture of breast implant surfaces is a critical factor in modulating the immune response in the periprosthetic environment.

## Results

**Figure S1.**
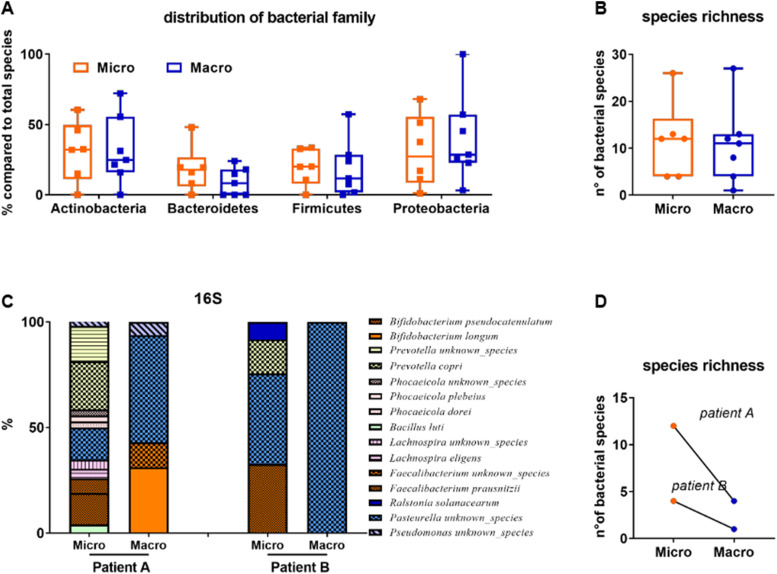
Taxonomic analysis of bacteria in periprosthetic fluids derived from patients with microtextured (Micro) or macrotextured (Macro) implants assessed through shotgun metagenomic sequencing. **(A, B)** Distribution of bacterial families and bacterial species richness found in the periprosthetic fluid. **(C, D)** Comparison between microbiota of same patients implanted with different textured prosthesis in terms of taxonomic analysis and species richness.

Periprosthetic fluids collected during the removal of both microtextured and macrotextured breast implants were first analyzed for bacterial contamination through a shotgun metagenomic approach (Fig S1; see the Materials and Methods section). The overall bacterial load in the periprosthetic fluids of our patient pool was generally low. However, the bacterial families identified, which included Actinobacteria, Bacteroides, Firmicutes, and Proteobacteria, demonstrated a similar richness of species across both types of implant surfaces (Fig S1A and B). Predominantly, species from the *Bifidobacterium* genus were identified, which are also found in breast milk (Yan et al, 2021). This may suggest a potential link between the mammary glands and the prosthesis pocket. Notably, in two patients having bilateral prostheses with different surface topographies, a reduction in the number of bacterial species was observed in the macrotextured implant compared with the microtextured one (Fig S1C and D).

**Figure 1.**
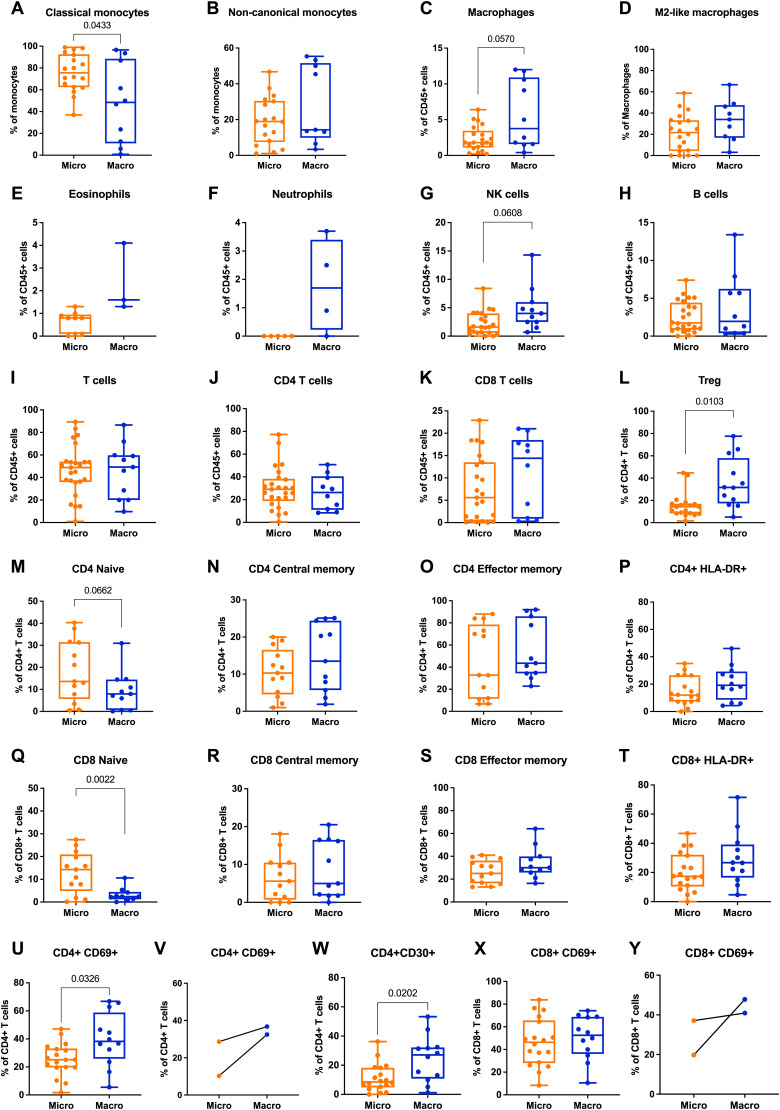
Flow cytometry analysis of periprosthetic leukocytes from patients with microtextured and macrotextured breast prostheses. Frequencies of the different immune subsets calculated as **(A, B)** frequency of viable monocytes, **(C, E, F, G, H, I, J, K)** frequency of viable CD45+ cells, (D) frequency of viable macrophages, **(L, M, N, O, P, U, V, W)** frequency of viable CD4+ T cells, **(Q, R, S, T, X, Y)** frequency of viable CD8+ T cells. Each histogram in the figure represents the mean ± SD of the measured parameters. The statistical significance of differences between microtextured and macrotextured prostheses was determined using an unpaired *t* test with Welch’s correction.

We then performed a multiparametric fluorescence-activated cell sorting (FACS) analysis on leukocytes derived from the periprosthetic fluid samples to investigate their composition and characteristics (see the Materials and Methods section). The frequency of classical monocytes, which exhibit an inflammatory phenotype and are characterized by CD14^+^/CD16^−^ markers, was found to be decreased in fluids associated with macrotextured implants compared with those with microtextured surfaces (Fig 1A and B). Conversely, in macrotextured implants, we observed an increase, albeit not statistically significant, in macrophages, particularly the CD163^+^CD206^+^ subset which is known for its immunosuppressive properties (Fig 1C and D). Further analysis of the immune system components showed an increasing trend in the percentage of eosinophils, neutrophils, natural killer (NK) cells, and CD8 cytotoxic T lymphocytes and a statistically significant increase in T-regulatory (Treg) cells—indicative of an immunosuppressive microenvironment and often found in tumors—among CD45^+^ cells in the periprosthetic fluids (Fig 1E–L). T-cell profiling revealed notable differences in the maturation of T lymphocytes between macrotextured and microtextured implant groups. Specifically, we observed a decreasing trend in naive CD4^+^ T cells in the macrotextured group, which was paralleled by an increasing frequency of central memory (CM) and effector memory (EM) CD4^+^ T cells (Fig 1M–O). Similarly, in CD8^+^ T cells, we reported a statistically significant contraction in the naive subset (Fig 1Q–S), indicating a potential shift toward a more mature immune response. For T-cell activation analysis, we employed markers such as HLA-DR, CD69, and CD30 (Fig 1P, T, and U–Y), the latter being particularly relevant because of its association with BIA-ALCL (Wolfram et al, 2012). In the periprosthetic fluids collected from patients with macrotextured implants, a statistically significant increase was observed in the frequencies of CD69^+^ and CD30^+^ cells among CD4^+^ T cells, underscoring a heightened activation state (Fig 1P, T, U, W, and X). In two unique cases, who had bilateral implants with different textures, we noted a similar trend in the immune response (Fig 1V and Y).

**Figure 2.**
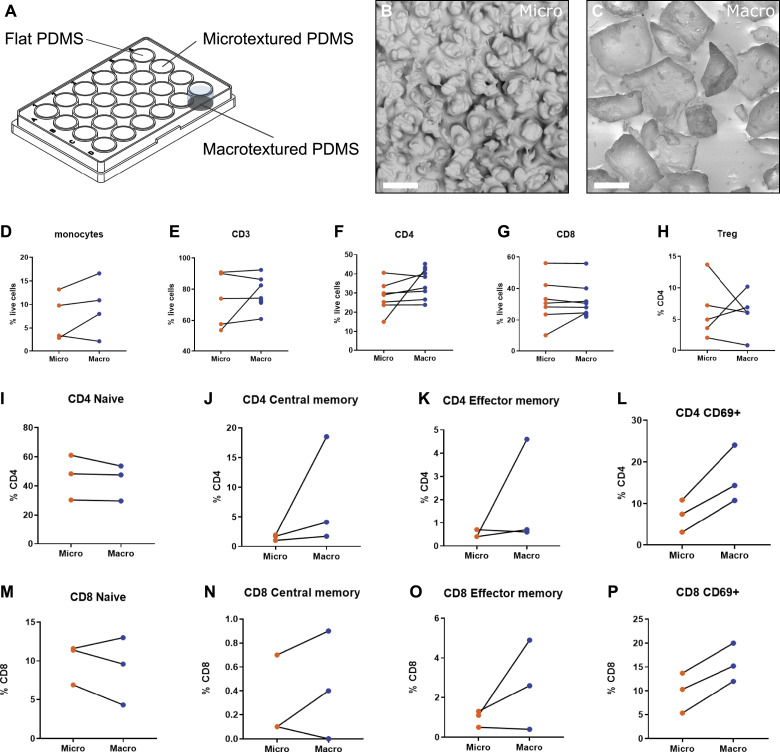
Flow cytometry analysis of PBMCs cultured on model surface textures. **(A)** Schematic representation of the in vitro experimental model used to assess the immune response of PBMCs to different surface textures. **(B)** Scanning electron microscopy image of a PDMS replica of a microtextured surface (Mentor Siltex), showing rounded bumps and cavities, with dimensions ranging from 10 to 50 μm, created using the coating emulation technique. **(C)** Scanning electron microscopy image of a PDMS replica of a macrotextured surface (Allergan Biocell), showing cubic cavities characteristic of this texture, with dimensions ranging from 100 to 400 μm, obtained by the “salt loss” technique. **(D, E, F, G, H, I, J, K, L, M, N, O, P)** Results of flow cytometry analysis depicting various immune cell populations and their activation status in PBMCs cultured on these model surfaces.

**Figure S2.**
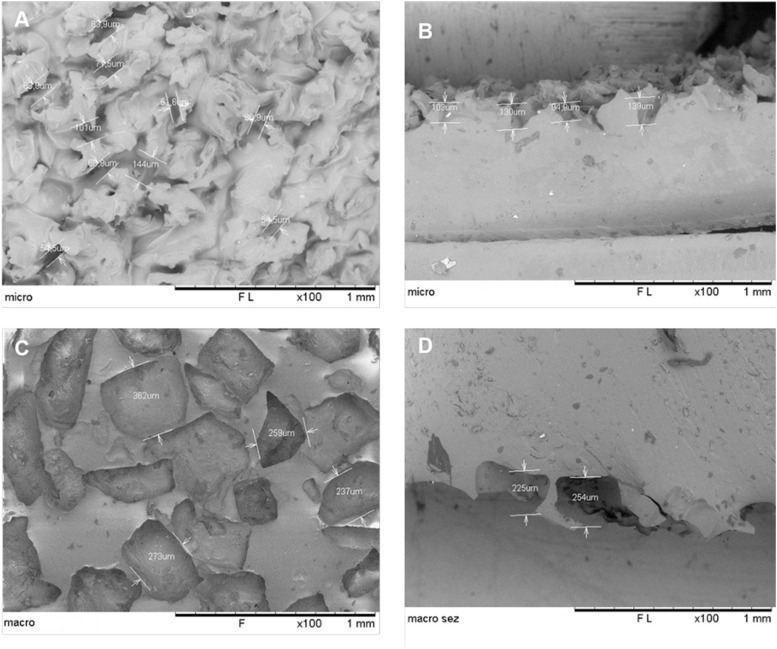
Topographic features of model surface replicas. **(A)** Scanning electron microscopy (SEM) image of a polydimethylsiloxane (PDMS) model of a microtextured surface showing measurements of topographic features. **(B)** Cross section of a microtextured model surface. **(C)** SEM image of the PDMS model of a macrotextured surface showing measurements of topographic features. **(D)** Cross section of a macrotextured model surface.

**Figure S3.**
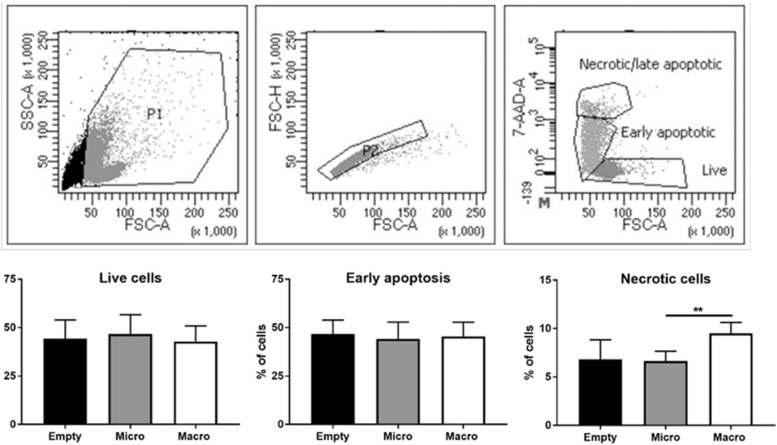
Gating strategy applied for assessing PBMC viability by flow cytometry. Physical parameters were used to sequentially exclude debris and doublets from the analysis. On singlets, PBMCs were defined alive based on 7AAD fluorescence. A representative analysis is shown in the upper row. In vitro cell viability assays: live, early apoptotic, and necrotic cells were analyzed (lower row). Histograms represent the mean ± SD. The statistical significance was determined using an unpaired *t* test with Welch’s correction. **P < 0.001.

Motivated by our ex vivo findings, we developed an in vitro platform to investigate the immediate immune response to specific breast implant surface topographies. This approach allowed us to study cellular reactions in a controlled environment, free from the confounding factors present in ex vivo samples, such as genetic backgrounds, patient histories, and variations in the duration of implantation. To mimic real-world conditions, we replicated the exact topographies of commercial microtextured and macrotextured breast implants (Figs 2A–C and S2A–D) using polydimethylsiloxane (PDMS). This material was chosen for its similarity to the silicone used in actual breast implants and for its biocompatibility, non-toxicity, and ease of casting (see the Materials and Methods section). Human PBMCs from healthy female donors were cultured on microtextured and macrotextured PDMS surfaces for 48 h without any vital stimuli, after which they were analyzed via FACS to assess viability and immune activation (see the Materials and Methods section, Figs 2 and S3). Although the macrotextured surfaces were associated with a marginally higher fraction of necrotic cells compared with the microtextured ones, the percentage of viable cells observed in the presence of both microtextured and macrotextured surfaces were similar to those observed in the flat, empty control wells (Fig S3).

No significant differences were observed in the frequencies of monocytes and T cells—including CD4 and CD8 subsets and CD4 Treg—between PBMCs cultured on microtextured and macrotextured surface replicas (Fig 2D–H). Notably, the in vitro model revealed an increase in effector and central memory cells for CD4 (Fig 2J and K) and CD8 (Fig 2N and O) on macrotextured surfaces, in agreement with our ex vivo results. Meanwhile, naive CD4 did not display any variation (Fig 2I), whereas naive CD8 were reduced on macrotextured surfaces (Fig 2M). In addition, activated CD4 and CD8 cells, marked by CD69 expression, were increased in PBMCs cultured on macrotextured surfaces (Fig 2L and P). This finding aligns with the ex vivo observations (Fig 1U, V, X, and Y).

**Figure 3.**
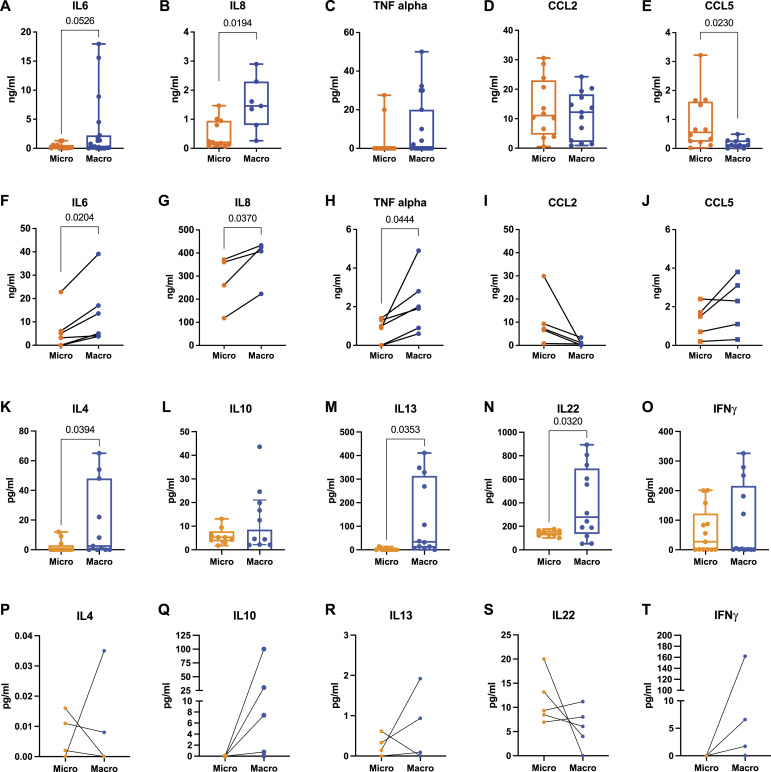
Quantification of soluble mediators in periprosthetic fluids and supernatants of PBMCs cultured on microtextured or macrotextured surfaces. **(A, B, C, D, E)** ELISA quantification of IL6, IL8, TNF-alpha, CCL2, and CCL5 in the periprosthetic fluid of patients with either microtextured or macrotextured implants. **(F, G, H, I, J)** ELISA quantification of the same set of cytokines and chemokines (as in (A, B, C, D, E)) released from PBMCs cultured on microtextured or macrotextured surfaces. **(K, L, M, N, O)** ELLA quantification of IL4, IL10, IL13, IL22, and INFɣ in the periprosthetic fluid of patients with microtextured and macrotextured implants. **(P, Q, R, S, T)** ELLA quantification of the same set of cytokines (as in (F, G, H, I, J)) released from PBMCs cultured on microtextured or macrotextured surfaces. Histograms represent the mean ± SD, based on data from at least four independent donors. **(A, B, C, D, E, F, G, H, I, J, K, L, M, N, O, P, Q, R, S, T)** Statistical significance was determined using an unpaired *t* test with Welch’s correction (A, B, C, D, E, K, L, M, N, O) and a paired *t* test (F, G, H, I, J, P, Q, R, S, T).

**Figure S4.**
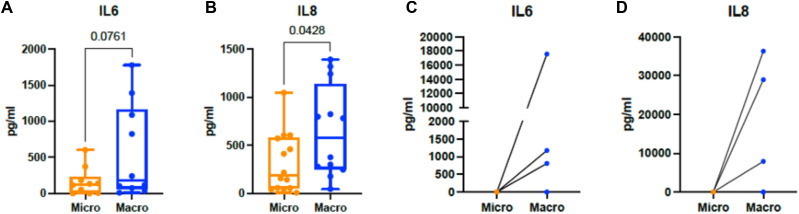
ELLA quantification of IL6 and IL8. **(A, B)** Cytokine levels in periprosthetic fluid derived from patients with microtextured (Micro) or macrotextured (Macro) implants. Histograms represent the mean ± SD. Statistical significance was assessed using an unpaired *t* test with Welch’s correction. **(C, D)** Cytokine levels in supernatants of PBMCs cultured on model surface textures.

To ascertain whether the periprosthetic microenvironment in patients is influenced by the texture of implant surfaces, we conducted an extensive cytokine analysis. This included pro-inflammatory cytokines such as IL6 and IL8, typically elevated in the tumor microenvironment, and TNF-alpha, known for its association with T-cell activation. We also analyzed the chemokines CCL2 and CCL5, which are known for their chemoattractant properties for immune cells. Enzyme-linked immunosorbent assay (ELISA) analysis of periprosthetic fluids revealed significantly elevated levels of IL6, IL8, and TNF-alpha in macrotextured implants compared with microtextured ones (Fig 3A–C). Although CCL2 levels were comparable between the two groups, a notable reduction in CCL5 was observed in the macrotextured group (Fig 3D and E). These findings, particularly for IL6 and IL8, were further validated using ELLA technology. ELLA’s high sensitivity and capacity for simultaneous multiple immunoassays confirmed these results (Fig S4A and B). In addition, we expanded our analysis using ELLA to include cytokines previously associated with BIA-ALCL, such as IL4, IL10, IL13, IL22, and INFɣ (Turner et al, 2020; Wang et al, 2021; Zhang et al, 2022). This analysis showed significantly higher levels of IL4, IL13, and IL22 in the periprosthetic fluid from the macrotextured group compared with the microtextured group (Fig 3K–O), highlighting a distinct cytokine profile potentially relevant to the pathogenesis of ALCL.

Consistent with these findings, in vitro ELISA analysis revealed that PBMCs cultured on macrotextured model surfaces secreted significantly higher levels of IL6, IL8, and TNF-alpha compared with those on microtextured surfaces (Fig 3F–H). Although the levels of CCL5 remained consistent across both types of surfaces, CCL2 was found to be lower in the culture supernatants from PBMCs cultured on macrotextured surfaces (Fig 3I and J). These results align with the ELLA findings from culture supernatants, which corroborate the ELISA data for IL6 and IL8 (Fig S4C and D). In addition, further ELLA analysis of cytokines previously linked to ALCL showed that with the exception of IL22, cytokine levels were elevated in supernatants from PBMCs cultured on macrotextured surfaces compared with those on microtextured ones (Fig 3F–H and P–T). This comprehensive dataset suggests that macrotextured surfaces are more prone to activating immune cell responses, thereby promoting an inflammatory, chronic-like microenvironment.

**Figure 4.**
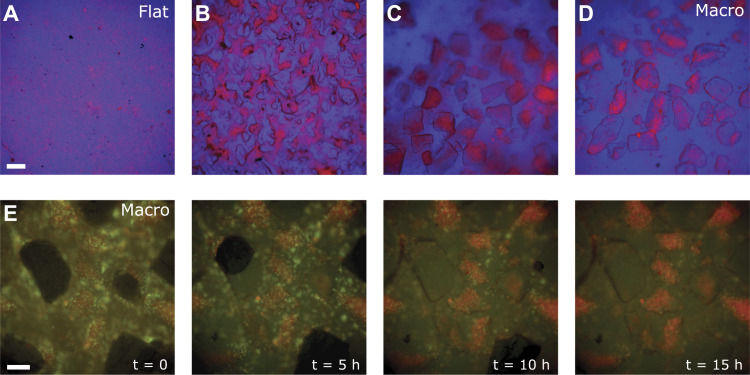
Leukocyte distribution on model surface textures. **(A, B, C, D)** Overlay of phase-contrast (blue) and fluorescent images (red indicating PBMCs) acquired 12 h after plating cells on different surfaces: flat (A), microtextured (B), and macrotextured ((C) for donor 1, (D) for donor 2). Scale bar, 200 μm. **(E)** Overlay of phase-contrast and fluorescent images (green for monocytes, red for lymphocytes) acquired at intervals of 0, 5, 10, and 15 h from the beginning of the experiment. Here, cells were plated on a macrotextured surface and co-cultured with *S. epidermidis*. Scale bar, 100 μm.

To elucidate the potential mechanisms underlying leucocyte activation on macrotextured surfaces, we analyzed the distribution of PBMCs, marked with a vital fluorescent label, across different surface textures over time (see the Materials and Methods section). On flat surfaces, PBMCs showed a random distribution (Fig 4A). In contrast, a distinct pattern was observed with microtextured samples (Fig 4B), becoming even more pronounced for macrotextured samples, where PBMCs accumulated within the characteristic surface cavities (Fig 4C and D). This behavior suggests a specific interaction of PBMCs with the varying textures, highlighting the role of surface topography in influencing cellular distribution. Distinguishing lymphocytes (red fluorochrome) from monocytes (green fluorochrome) allowed us to further observe distinct behaviors on these surfaces. Specifically, lymphocytes were seen progressively moving into and accumulating within the cavities of the macrotextured surfaces (Fig 4E). In contrast, monocytes displayed greater mobility and were predominantly found outside these cavities (Video 1). This behavior was consistent under sterile conditions (Fig 4A and D) and when surfaces were pre-treated with a diluted concentration of *Staphylococcus epidermidis* (Caldara et al, 2022)—a common culprit in breast implant–associated infections—24 h before cell plating (Fig 4E). The unchanged behavior in the presence of bacteria further emphasizes that the response is driven by surface texture rather than microbial factors. These observations suggest that the activation of lymphocytes on macrotextured surfaces may be significantly influenced by their entrapment within surface cavities. Factors such as the localized concentration of soluble mediators and cell density could be pivotal in driving this activation, highlighting the complex interplay between physical surface features and cellular responses in the immune system.

Video 1 Overlay of phase-contrast and fluorescent images (green for monocytes, red for lymphocytes) acquired every 10 min with cells plated on a macrotextured surface and co-cultured with *S. epidermidis*.Download video

## Discussion

BIA-ALCL emerges as a complex condition, influenced by genetic predispositions, bacterial biofilms, chronic inflammation, and textured breast implants’ properties, indicating a multifaceted pathogenesis (Turner et al, 2020; Wang et al, 2021). In this work, although we did not perform a genetic analysis of our patients’ backgrounds, the evidence suggests that genetic factors alone may not fully explain the observed differences in immune response. This inference is drawn from two key observations in our study. First, our in vitro results were derived from culturing cells from healthy donors on well plates with different surface textures. The observed differences in response across these textures hint at the influence of the implant’s surface characteristics over a genetic predisposition in these controlled conditions. Second, in two unique cases, we analyzed cells from the periprosthetic fluid of patients with bilateral implants of different textures. The variance in response between these two implants within the same patient again points toward the impact of the implant’s texturization. Although these observations indicate that the texture of implants plays a significant role in leukocyte activation, we acknowledge that they do not entirely rule out the influence of genetic factors. Future studies involving a comprehensive genetic analysis could provide more definitive insights into the interplay between the genetic background and implant texture in modulating immune responses.

The findings from our investigation, including the low bacterial load and the nature of the identified species, challenge the assumption that bacterial biofilms are the primary driver of the chronic inflammatory state linked to macrotextured breast implants. Notably, our experiments showed no significant difference in lymphocyte accumulation on macrotextured surfaces, both in the absence and presence of bacteria. These observations are particularly relevant considering the established correlation between biofilm formation and capsular contraction, which is thought to increase the risk of developing ALCL (Alessandri-Bonetti et al, 2023). Although the presence of bacteria has been a focal point in understanding the etiology of implant-associated complications, our results suggest that the physical properties of the implant surface itself may play a more pivotal role in initiating and sustaining chronic inflammatory responses.

Our study reveals that macrotextured surfaces are associated with elevated levels of pro-inflammatory cytokines IL6, IL8, and TNF-alpha in periprosthetic fluids from patients, pointing to an enhanced inflammatory response linked to the implants’ specific topography. Furthermore, our cytokine profile analysis, focusing on markers like IL4, IL10, IL13, IL22, and INFɣ previously linked to BIA-ALCL, has identified increased levels in association with macrotextured surfaces. Moreover, we observed a heightened activation in both the CD8 and CD4 compartments on macrotextured surfaces, characterized by an increased presence of effector and central memory cells, alongside noteworthy elevations in Treg and CD69^+^ cells, compared with microtextured ones. Particularly significant is the observed predominance of CD30^+^ cells in the periprosthetic fluid of patients with macrotextured implants. Considering BIA-ALCL’s hallmark association with CD30^+^ markers (Quesada et al, 2019; Zhang et al, 2022), these results underscore a crucial link between implant surface texture and an immunological milieu that may predispose to lymphomas associated with implantable devices.

The observed higher prevalence of tumor-associated macrophages—typically marked by CD206 and CD163 and known for their immunosuppressive behavior (Belgiovine et al, 2020)—in the collected samples from patients with macrotextured implants further suggests that the polarization of macrophages may be influenced by the implant’s surface topography. Such macrophages are implicated in promoting tumor development and survival and resistance to conventional antitumor treatments (Germano et al, 2011; De Palma & Lewis, 2013). In line with findings in BIA-ALCL tissues (Laurent et al, 2016), our study also indicates an increasing trend in NK cells, eosinophils, and neutrophils in the context of macrotextured prostheses. This points to a more pronounced inflammatory niche associated with these implants, potentially contributing to the chronic-like inflammatory environment observed. These comprehensive findings underscore the critical role of implant surface texture in shaping the immune response, possibly influencing the risk and progression of conditions such as BIA-ALCL.

The development of reliable and reproducible physical models for in vitro testing is crucial for conducting extensive, long-term experimental analyses of the biological effects of implanted prostheses. Such models offer an invaluable platform for investigating the direct mechanisms that may lead to possible adverse events. Factors such as surface area, roughness, the depth of cavities and pores, and the structured of edges have been previously indicated as significant contributors to the body’s reactions to implants (Barr et al, 2017; Loch-Wilkinson et al, 2020; Belgiovine et al, 2023). Our in vitro cultures using PBMCs on the model surfaces created for this study not only demonstrated the biocompatibility of the material used (PDMS) but also provided insights into the cellular responses to different surface topographies. The cytokine release patterns observed in these in vitro experiments were consistent with those seen in ex vivo samples from patients. This consistency strengthens the relevance of our model for mechanistic studies, highlighting its capability to accurately replicate the biological interactions occurring in the body postimplantation.

We observed that leukocytes, particularly lymphocytes, tend to be captured within the cavities of macrotextured surfaces. This entrapment appears to stimulate these cells to release inflammatory cytokines, a finding in line with recent evidence linking lymphomas with implantable devices (Brody, 2016). In addition, the concept of “tribology”—the study of friction between interacting surfaces—is gaining attention in the context of implant carcinogenicity, as seen in orthopedic implants (Clemens et al, 2019). This aspect of physical interaction between implant surfaces and biological tissues offers a new perspective on the mechanisms underlying chronic inflammation. Previously, it was hypothesized that chronic inflammation might be linked to the aging and wear of silicone prostheses, suggesting that silicone itself could trigger an inflammatory reaction (Bizjak et al, 2015). However, our findings indicate that the topographical features of the implant surface, rather than the silicone material per se, might play a more significant role in initiating and sustaining inflammatory reactions.

The impact of surface topography on implant-associated inflammation is increasingly being recognized as a critical factor in the establishment of a pro-tumoral environment, potentially leading to conditions like BIA-ALCL. Consequently, the development of less irritating and more inert implant surfaces may hold promise for improving the safety and efficacy of prosthetic devices across various medical fields. Given the potential health implications, ongoing research in this area is paramount importance. Recent studies have expanded the correlation between lymphomas and implantable devices beyond breast prostheses. Lymphomas have been linked to a range of devices, including cardiac, joint, gluteal, testicular, and intraocular implants, made from both silicone and non-silicone materials (Moruzzo et al, 2009; Palraj et al, 2010; Sanchez-Gonzalez et al, 2013; Kellogg et al, 2014; Vivacqua et al, 2015). Although genetic predisposition plays a role, it is evident that implants themselves can influence the inflammatory response in the body. Moreover, concerns extend beyond lymphomas. Studies have shown associations between breast implants and autoimmune diseases and a heightened incidence of breast cancer recurrence in patients who underwent heterologous reconstruction postmastectomy (Watad et al, 2018; Lee et al, 2020; Tervaert et al, 2022). Our findings underscore the importance of surface texture in eliciting immune responses and suggest that the chronic inflammation observed may be more directly attributable to the physical characteristics of these implants than previously understood, potentially increasing the oncological risk for patients.

Although our study provides crucial insights into the immune responses triggered by different breast implant textures, we recognize the limitations inherent in our research, especially when considering a condition as rare as BIA-ALCL. This challenge is compounded by the difficulty in assembling a large, diverse sample pool, which impacts the statistical robustness and limits the scope of our conclusions. In our cohort of patients, we did not encounter any cases of BIA-ALCL. However, the observed increase in cytokines such as IL10 and IL13 in fluids from patients with macrotextured implants, and similar findings in our in vitro model, may hint at a potential dysregulation associated with these implant textures. Yet, we must exercise caution when extrapolating these results to the specific context of BIA-ALCL. Further research, involving a larger and more diverse sample population, is crucial to validate our findings within the unique pathology of BIA-ALCL. We also acknowledge the presence of potential biases in our study, including selection bias because of our choice of specific implant types and textures and observer bias in data interpretation. We have endeavored to mitigate these biases through careful experimental design, data collection, and analysis, yet complete elimination of these biases is challenging.

This research represents an initial exploration into a complex and evolving field. It sets the stage for future investigations, underscoring the need for more expansive and diverse studies to fully understand the interactions between breast implants and the immune system. Prospective studies, in particular, are essential to provide a deeper assessment of the long-term effects of implant surface textures on immune responses. Such studies hold the potential to guide the design of safer and more biocompatible prosthetic devices, ultimately improving patient outcomes. Our findings, therefore, not only contribute to the existing body of knowledge but also open avenues for further research that can significantly impact the field of implantable medical devices.

## Materials and Methods

### Patients’ selection

We enrolled in the study 43 patients who had an intact breast implant (including both breast prostheses and expanders), with the criteria that these implants were neither exposed to the external environment nor infected. These encompassed cases of both breast prosthetic replacement (for esthetic or reconstructive reasons) and second-stage breast reconstruction. A total of 53 breasts were collected; of these, 24 breasts (45.3%) had macrotextured implants, whereas the remaining 29 breasts (54.7%) were fitted with microtextured devices. Notably, 34 of the 53 breast samples (64.2%) were collected from patients with a history of breast cancer. Radiotherapy was administered in 11 out of the 53 cases (20.7%). In terms of implant duration, only samples that had been in place for a minimum duration of 6 mo were included. In particular, 34 implants (64.2%) were in place for a period ranging from 6 to 24 mo, whereas the remaining 19 implants (35.8%) were retained for over 2 yr. All patients were provided with written informed consent which was signed before surgery. The study was approved by the Local Ethics Committee (reference number 163/21; CE Humanitas). Detailed clinical data of patients enrolled are reported in Table S1.

Table S1 Clinical data of the patients enrolled in the study.

### Sample collection

In all enrolled subjects, the periprosthetic fluid was collected using a 10-ml sterile syringe immediately after making an incision of the capsule. The collected fluid was then promptly transferred to a sterile container for analysis. For shotgun metagenomic analysis, an aliquot of the collected periprosthetic fluid was stored at −80°C immediately after collection. For the evaluation of the immune landscape, collected periprosthetic fluid samples were centrifuged for 10 min at 780*g* to separate cells from the liquid phase. Cells were analyzed by flow cytometry, whereas the liquid phase was used to measure the soluble molecules by ELISA and ELLA, as described below.

### Shotgun metagenomic analysis

Microbial DNA was extracted from 20 periprosthetic fluid samples using a commercial, ultrasensitive kit (Ultra-Deep Microbiome Prep; Molzym GmbH). However, seven of these samples were found to have DNA concentrations too low and were subsequently excluded from the shallow shotgun profiling that was performed by GenProbio. Out of 13 samples analyzed, 7 were associated with macrotextured implants and 6 with microtextured implants. This sample set included two patients with bilateral prostheses, each featuring different surface topographies.

### Ex vivo flow cytometry analysis

Flow cytometry was performed after standard procedures (Cossarizza et al, 2021) on cells derived from periprosthetic fluids collected from 16 patients with macrotextured implants and 27 patients with microtextured implants. FACS analysis was not carried out for all samples because some samples were inadequate, in terms of quantity or contamination. Samples were stained with a live-dead exclusion dye (LIVE/DEAD Fixable Aqua Dead Cell Stain Kit) for 15 min at RT to discriminate dead and viable cells. Subsequently, cells were incubated with the monoclonal antibodies listed in Table S2 for surface antigen staining. After the staining, labeled cells were fixed in PBS + 1% formalin. The acquisition was performed at FACSymphony A5 (BD Biosciences). FACS data were analyzed with FlowJo X 10.0.7r2 software (BD).

Table S2 Monoclonal antibodies for surface antigen staining used in the study.

### In vitro flow cytometry analysis

We used human PBMCs isolated from the buffy coats of healthy female donors. The PBMCs were separated using a Histopaque-1077 gradient (Sigma-Aldrich) and then cultured on model surface with different textures, previously exposed to UV light for sterilization. PBMCs were plated at a concentration of 2 × 10^6^ cells per well and cultured for 48 h at 37°C with 5% CO_2_ in 24-well microplates (Costar; Corning Incorporated). After 48 h of culture on texture surface replicas, PBMCs were collected for FACS analysis. Cell viability was assessed using 7AAD staining (BD Biosciences), following the manufacturer’s instructions. To assess identity immune cell populations and evaluate their activation state, cells were incubated with the monoclonal antibodies listed in Table S2 for surface antigen staining. After the staining, labeled cells were fixed in PBS + 1% formalin. The acquisition was performed at FACSymphony A5 (BD Biosciences). A minimum of 50,000 events were acquired for each sample. Data were analyzed using BD FACSDiva 8.0.1 software (BD Biosciences) and FlowJo X 10.0.7r2 software (BD).

### Model surface preparation

Model surfaces made of silicone elastomer were prepared to replicate microtextured and macrotextured implants. For macrotextured surfaces, we employed the “salt loss” technique, whereas a double replication process was used for microtextured surfaces. For this purpose, we selected SYLGARD 184 (Dow Corning), a low-viscosity, transparent PDMS polymer, chosen for its biocompatibility, non-cytotoxicity, and excellent flow properties (Rusconi et al, 2014). These characteristics are crucial for accurate surface replication and easy inspection of the final components. The viscosity of SYLGARD 184 was monitored during curing to evaluate the evolution of its flow properties and determine the optical gelation time. Replicas of microtextured and macrotextured surfaces were created by pouring and curing the elastomer in polystyrene wells under identical conditions as those employed for the textured models (Figs 2 and S2). In addition, to provide a baseline comparison, we fabricated non-textured control surfaces using the same silicone rubber.

### Diagnostic assays

The expression levels of the analyzed soluble molecules were investigated in both periprosthetic fluids and in culture supernatants by ELISA and ELLA. Periprosthetic samples were processed as previously described. Supernatant fluids were collected after 48 h of culture and centrifuged at 285*g* for 5 min before proceeding with the analysis. For the quantification of human cytokines IL-6, IL-8, TNF-alpha, CCL2, and CCL5, we used commercial ELISA kits (R&D Systems) according to the manufacturer’s guidelines. The data obtained from these assays were subsequently analyzed using SoftMax Pro 5.3 software. The ELLA system (ProteinSimple; Bio-Techne) for automated enzyme-linked immunoassays of the cytokines IL4, IL6, IL8, IL10, IL13, IL22, and INFɣ. ELLA, based on microfluidic technology, allows for the performance of these assays with minimal manual intervention. Samples were diluted 1:1 with the washing buffer and pipetted into the instrument’s cartridge. Each cartridge comes with a pre-generated calibration curve by the manufacturer for each lot, and the ELLA system reads the cartridge’s barcode to acquire calibration-related parameters. The quantification of cytokines was then performed based on these master calibration curves, with fluorescent signals being read and processed internally by the ELLA instrument.

### Microscopy experiments

PDMS surface replicas mimicking various textures, placed in the bottom of a polystyrene 24-well chamber, were first sterilized under UV light. PBMCs, pre-stained with CellTracker Fluorescent Probes (Thermo Fisher Scientific Inc.), were seeded onto these surfaces. In experiments involving bacterial interaction, a suspension of *S. epidermidis* (strain ATCC 12228, OD = 0.1) in Tryptone Broth was added to the wells and incubated for 24 h at 37°C, after which the bacterial medium was replaced with the PBMC solution. Imaging was performed on a DMI8 Leica microscope, utilizing a 20× air objective and maintained under climate control at 37°C. For each experimental condition, 10 images across different vertical planes were captured using an ORCA-Flash 4.0 V3 Digital CMOS camera (Hamamatsu). Time-lapse imaging was conducted at 10-min intervals for up to 5 h, employing Metamorph (v7.10.1.161) for image acquisition.

### Statistical analysis

Statistical analysis was conducted using an unpaired *t* test with Welch’s correction or paired *t* test, as indicated (GraphPad 9, Prism statistical package). Continuous variables, encompassing the data derived from cytokine quantification and cell behavior analysis, are presented as mean ± SD. A *P*-value of less than 0.05 was set to determine statistical significance.

## Supplementary Material

Reviewer comments

## References

[bib1] Alessandri-Bonetti M, Jeong T, Vaienti L, De La Cruz C, Gimbel ML, Nguyen VT, Egro FM (2023) The role of microorganisms in the development of breast implant-associated anaplastic large cell lymphoma. Pathogens 12: 313. 10.3390/pathogens12020313 36839585 PMC9961223

[bib2] Arber DA, Orazi A, Hasserjian R, Thiele J, Borowitz MJ, Le Beau MM, Bloomfield CD, Cazzola M, Vardiman JW (2016) The 2016 revision to the World Health Organization classification of myeloid neoplasms and acute leukemia. Blood 127: 2391–2405. 10.1182/blood-2016-03-643544 27069254

[bib3] Barnsley GP, Sigurdson LJ, Barnsley SE (2006) Textured surface breast implants in the prevention of capsular contracture among breast augmentation patients: A meta-analysis of randomized controlled trials. Plast Reconstr Surg 117: 2182–2190. 10.1097/01.prs.0000218184.47372.d5 16772915

[bib4] Barr S, Hill EW, Bayat A (2017) Functional biocompatibility testing of silicone breast implants and a novel classification system based on surface roughness. J Mech Behav Biomed Mater 75: 75–81. 10.1016/j.jmbbm.2017.06.030 28697402

[bib5] Belgiovine C, Digifico E, Anfray C, Ummarino A, Torres Andón F (2020) Targeting tumor-associated macrophages in anti-cancer therapies: Convincing the traitors to do the right thing. J Clin Med 9: 3226. 10.3390/jcm9103226 33050070 PMC7600332

[bib6] Belgiovine C, Pellegrino L, Bulgarelli A, Lauta FC, Di Claudio A, Ciceri R, Cancellara A, Calcaterra F, Mavilio D, Grappiolo G, (2023) Interaction of bacteria, immune cells, and surface topography in periprosthetic joint infections. Int J Mol Sci 24: 9028. 10.3390/ijms24109028 37240374 PMC10218985

[bib7] Bewtra C, Gharde P, Dharmashi J (2022) Current understanding of breast implant-associated anaplastic large cell lymphoma. Cureus 14: e30516. 10.7759/cureus.30516 36420249 PMC9678239

[bib8] Bizjak M, Selmi C, Praprotnik S, Bruck O, Perricone C, Ehrenfeld M, Shoenfeld Y (2015) Silicone implants and lymphoma: The role of inflammation. J Autoimmun 65: 64–73. 10.1016/j.jaut.2015.08.009 26330346

[bib9] Brody GS (2016) The case against biofilm as the primary initiator of breast implant–associated anaplastic large cell lymphoma. Plast Reconstr Surg 137: 766e–767e. 10.1097/01.prs.0000480003.80422.03 26809044

[bib10] Caldara M, Belgiovine C, Secchi E, Rusconi R (2022) Environmental, microbiological, and immunological features of bacterial biofilms associated with implanted medical devices. Clin Microbiol Rev 35: e0022120. 10.1128/cmr.00221-20 35044203 PMC8768833

[bib11] Clemens MW, Brody GS, Mahabir RC, Miranda RN (2018) How to diagnose and treat breast implant–associated anaplastic large cell lymphoma. Plast Reconstr Surg 141: 586e–599e. 10.1097/prs.0000000000004262 29595739

[bib12] Clemens M, DeCoster R, Fairchild B, Bessonov A, Santanelli di Pompeo F (2019) Finding consensus after two decades of breast implant-associated anaplastic large cell lymphoma. Semin Plast Surg 33: 270–278. 10.1055/s-0039-1696998 31632211 PMC6797486

[bib13] Cossarizza A, Chang H, Radbruch A, Abrignani S, Addo R, Akdis M, Andrä I, Andreata F, Annunziato F, Arranz E, (2021) Guidelines for the use of flow cytometry and cell sorting in immunological studies (third edition). Eur J Immunol 51: 2708–3145. 10.1002/eji.202170126 34910301 PMC11115438

[bib14] Davis C, Boyd C, Mateo de Acosta Andino DA, Kumbla PA, Sanchez RJ, Kurapati S, King TW, de la Torre JI (2020) Dermal autografts in breast reconstruction: A review of past and current trends. Ann Plast Surg 84: 618–622. 10.1097/sap.0000000000002128 31904644

[bib15] De Palma M, Lewis CE (2013) Macrophage regulation of tumor responses to anticancer therapies. Cancer Cell 23: 277–286. 10.1016/j.ccr.2013.02.013 23518347

[bib16] Doloff JC, Veiseh O, de Mezerville R, Sforza M, Perry TA, Haupt J, Jamiel M, Chambers C, Nash A, Aghlara-Fotovat S, (2021) The surface topography of silicone breast implants mediates the foreign body response in mice, rabbits and humans. Nat Biomed Eng 5: 1115–1130. 10.1038/s41551-021-00739-4 34155355

[bib17] Germano G, Mantovani A, Allavena P (2011) Targeting of the innate immunity/inflammation as complementary anti-tumor therapies. Ann Med 43: 581–593. 10.3109/07853890.2011.595732 21756064

[bib18] Keech JA, Creech BJ (1997) Anaplastic T-cell lymphoma in proximity to a saline-filled breast implant. Plast Reconstr Surg 100: 554–555. 10.1097/00006534-199708000-00065 9252643

[bib19] Kellogg BC, Hiro ME, Payne WG (2014) Implant-associated anaplastic large cell lymphoma: Beyond breast prostheses. Ann Plast Surg 73: 461–464. 10.1097/sap.0b013e31827faff2 23722577

[bib20] Laurent C, Delas A, Gaulard P, Haioun C, Moreau A, Xerri L, Traverse-Glehen A, Rousset T, Quintin-Roue I, Petrella T, (2016) Breast implant-associated anaplastic large cell lymphoma: Two distinct clinicopathological variants with different outcomes. Ann Oncol 27: 306–314. 10.1093/annonc/mdv575 26598546 PMC4722894

[bib21] Laurent C, Nicolae A, Laurent C, Le Bras F, Haioun C, Fataccioli V, Amara N, Adélaïde J, Guille A, Schiano J-M, (2020) Gene alterations in epigenetic modifiers and JAK-STAT signaling are frequent in breast implant-associated ALCL. Blood 135: 360–370. 10.1182/blood.2019001904 31774495 PMC7059458

[bib22] Lee K-T, Kim S, Jeon B-J, Pyon JK, Mun G-H, Ryu JM, Lee SK, Yu J, Kim SW, Lee JE, (2020) Association of the implant surface texture used in reconstruction with breast cancer recurrence. Jama Surg 155: 1132–1140. 10.1001/jamasurg.2020.4124 33026424 PMC7542523

[bib23] Loch-Wilkinson A, Beath KJ, Magnusson MR, Cooter R, Shaw K, French J, Vickery K, Prince HM, Deva AK (2020) Breast implant-associated anaplastic large cell lymphoma in Australia: A longitudinal study of implant and other related risk factors. Aesthet Surg J 40: 838–846. 10.1093/asj/sjz333 31738381

[bib24] Mankowski P, Carr M, Cherukupalli A, Bovill E, Lennox P, Brown MH, Carr N (2022) The macrotextured implant recall: Breast implant–associated-anaplastic large cell lymphoma risk aversion in cosmetic and reconstructive plastic surgery practices. Aesthet Surg J 42: 1408–1413. 10.1093/asj/sjac158 35709374

[bib25] Marra A, Viale G, Pileri SA, Pravettoni G, Viale G, De Lorenzi F, Nolè F, Veronesi P, Curigliano G (2020) Breast implant-associated anaplastic large cell lymphoma: A comprehensive review. Cancer Treat Rev 84: 101963. 10.1016/j.ctrv.2020.101963 31958739

[bib26] Maxwell GP, Scheflan M, Spear S, Nava MB, Hedén P (2014) Benefits and limitations of macrotextured breast implants and consensus recommendations for optimizing their effectiveness. Aesthet Surg J 34: 876–881. 10.1177/1090820x14538635 25024450

[bib27] Mempin M, Hu H, Vickery K, Kadin ME, Prince HM, Kouttab N, Morgan JW, Adams WP, Deva AK (2021) Gram-negative bacterial lipopolysaccharide promotes tumor cell proliferation in breast implant-associated anaplastic large-cell lymphoma. Cancers 13: 5298. 10.3390/cancers13215298 34771464 PMC8582399

[bib28] Moruzzo D, Bindi M, Bongiorni MG, Castiglioni M (2009) A rare case of non-Hodgkin lymphoma in a pacemaker pocket. Leuk Lymphoma 50: 1384–1385. 10.1080/10428190903039990 19562613

[bib29] Palraj B, Paturi A, Stone RG, Alvarez H, Sebenik M, Perez MT, Bush LM (2010) Soft tissue anaplastic large T-cell lymphoma associated with a metallic orthopedic implant: Case report and review of the current literature. J Foot Ankle Surg 49: 561–564. 10.1053/j.jfas.2010.08.009 20870426

[bib30] Quesada AE, Medeiros LJ, Clemens MW, Ferrufino-Schmidt MC, Pina-Oviedo S, Miranda RN (2019) Breast implant-associated anaplastic large cell lymphoma: A review. Mod Pathol 32: 166–188. 10.1038/s41379-018-0134-3 30206414

[bib31] Rusconi R, Garren M, Stocker R (2014) Microfluidics expanding the frontiers of microbial ecology. Annu Rev Biophys 43: 65–91. 10.1146/annurev-biophys-051013-022916 24773019 PMC4076152

[bib32] Sanchez-Gonzalez B, Garcia M, Montserrat F, Sanchez M, Angona A, Solano A, Salar A (2013) Diffuse large B-cell lymphoma associated with chronic inflammation in metallic implant. J Clin Oncol 31: e148–e151. 10.1200/jco.2012.42.8250 23401446

[bib33] Srinivasa DR, Miranda RN, Kaura A, Francis AM, Campanale A, Boldrini R, Alexander J, Deva AK, Gravina PR, Medeiros LJ, (2017) Global adverse event reports of breast implant-associated ALCL: An international review of 40 government authority databases. Plast Reconstr Surg 139: 1029–1039. 10.1097/prs.0000000000003233 28157770

[bib34] Cohen Tervaert JW, Mohazab N, Redmond D, van Eeden C, Osman M (2022) Breast implant illness: Scientific evidence of its existence. Expert Rev Clin Immunol 18: 15–29. 10.1080/1744666x.2022.2010546 34882509

[bib35] Tevis SE, Hunt KK, Miranda RN, Lange C, Butler CE, Clemens MW (2019) Differences in human leukocyte antigen expression between breast implant–associated anaplastic large cell lymphoma patients and the general population. Aesthet Surg J 39: 1065–1070. 10.1093/asj/sjz021 30715139

[bib36] Turner SD, Inghirami G, Miranda RN, Kadin ME (2020) Cell of origin and immunologic events in the pathogenesis of breast implant–associated anaplastic large-cell lymphoma. Am J Pathol 190: 2–10. 10.1016/j.ajpath.2019.09.005 31610171 PMC7298558

[bib37] Vivacqua A, Kerwin KJ, Tubbs R, Roselli EE (2015) Lymphoma of prosthetic aortic graft presenting as recurrent embolization. Ann Thorac Surg 99: 306–307. 10.1016/j.athoracsur.2013.12.089 25555949

[bib38] Wang Y, Zhang Q, Tan Y, Lv W, Zhao C, Xiong M, Hou K, Wu M, Ren Y, Zeng N, (2021) Current progress in breast implant-associated anaplastic large cell lymphoma. Front Oncol 11: 785887. 10.3389/fonc.2021.785887 35070989 PMC8770274

[bib39] Watad A, Rosenberg V, Tiosano S, Cohen Tervaert JW, Yavne Y, Shoenfeld Y, Shalev V, Chodick G, Amital H (2018) Silicone breast implants and the risk of autoimmune/rheumatic disorders: A real-world analysis. Int J Epidemiol 47: 1846–1854. 10.1093/ije/dyy217 30329056

[bib40] Wolfram D, Rabensteiner E, Grundtman C, Böck G, Mayerl C, Parson W, Almanzar G, Hasenöhrl C, Piza-Katzer H, Wick G (2012) T regulatory cells and TH17 cells in peri–silicone implant capsular fibrosis. Plast Reconstr Surg 129: 327e–337e. 10.1097/prs.0b013e31823aeacf 22286447

[bib41] Yan W, Luo B, Zhang X, Ni Y, Tian F (2021) Association and occurrence of bifidobacterial phylotypes between breast milk and fecal microbiomes in mother–infant dyads during the first 2 Years of life. Front Microbiol 12: 669442. 10.3389/fmicb.2021.669442 34163448 PMC8215152

[bib42] Zhang X-R, Chien P-N, Nam S-Y, Heo C-Y (2022) Anaplastic large cell lymphoma: Molecular pathogenesis and treatment. Cancers 14: 1650. 10.3390/cancers14071650 35406421 PMC8997054

